# J-147 a Novel Hydrazide Lead Compound to Treat Neurodegeneration: CeeTox^™^ Safety and Genotoxicity Analysis

**DOI:** 10.4172/2155-9562.1000158

**Published:** 2013-07-25

**Authors:** Paul A. Lapchak, Rene Bombien, Padmesh S. Rajput

**Affiliations:** 1 Department of Neurology and Neurosurgery, Cedars-Sinai Medical Center, Los Angeles, USA; 2 Division of Cardiothoracic Surgery, Cedars-Sinai Medical Center, Los Angeles, USA; 3 Department of Neurology, Advanced Health Sciences Pavilion, Los Angeles, USA

## Abstract

J-147 is a broad spectrum neuroprotective phenyl hydrazide compound with significant neurotrophic properties related to the induction of brain-derived neurotrophic factor (BDNF). Because this molecule is pleiotropic, it may have substantial utility in the treatment of a wide range of neurodegenerative diseases including acute ischemic stroke (AIS), traumatic brain injury(TBI), and Alzheimer’s disease(AD) where both neuroprotection and neurotrophism would be beneficial. Because of the pleiotropic actions of J-147, we sought to determine the safety profile of the drug using multiple assay analysis.

For CeeTox analyses, we used a rat hepatoma cell line (H4IIE) resulted in estimated C_Tox_ value (i.e.: sustained concentration expected to produce toxicity in a 14 day repeat dosing study) of 90 μM for J-147. The CeeTox panel shows that J-147 produced some adverse effects on cellular activities, in particular mitochondrial function, but only with high concentrations of the drug. J-147 was also not genetoxic with or without Aroclor-1254 treatment.

For J-147, based upon extensive neuroprotection assay data previously published, and the CeeTox assay (C_Tox_ value of 90 μM) in this study, we estimated in vitro neuroprotection efficacy (EC_50_ range 0.06–0.115 μM)/toxicity ratio is 782.6–1500 fold and the neurotrophism (EC_50_ range 0.025 μM)/toxicity ratio is 3600, suggesting that there is a significant therapeutic safety window for J-147 and that it should be further developed as a novel neuroprotective-neurotrophic agent to treat neurodegenerative disease taking into account current National Institute of Neurological Disorders and Stroke (NINDS) RIGOR guidelines.

## Introduction

There have been many attempts to develop neuroprotective or neurotrophic drugs belonging to diverse structural classes to treat acute ischemic stroke (AIS), traumatic brain injury (TBI) and Alzheimer’s disease(AD), but many have failed to show significant short or long-term benefit to the patient, in particular in stroke patients [1–5], but this is also true for TBI [6–10] and AD patients [11–15]. Recently a new hydrazide compound with prominent neuroprotective and neurotrophic properties was described in the literature [16,17].

Schubert and colleagues have shown that the drug known as J-147 ((E)-N-(2,4-Dimethylphenyl)-2,2,2-trifluoro-N′-(3-methoxybenzylidene) acetohydrazide) is effective at protecting a variety of primary cells and cell lines from “toxic” insults recapitulating one or more processes associated with AIS, TBI or AD [18,19].

Since the development of novel strategies to treat neurodegenerative diseases is extremely important to society, due to the escalating incidence of neurodegenerative diseases patients in the USA and worldwide [20], we have developed a translational development standard operating development procedure (SOP), which includes multi-tier in vitro and in vivo testing for both efficacy, safety and genotoxicity [21–23]. Previous reports by Schubert et al. have provided limited safety data and efficacy for J-147 to treat AD [16,17].

Based upon extensive in vitro analysis [16,17], J-147 has been shown to promotes HT22 and primary cell survival in a dose-dependent manner (EC_50_ value range of 0.06 – 0.115 μM) when applied to cell exposed to the mitochondrial neurotoxin, iodoacetic acid (IAA), toxicity mediated by the excitatory amino acid glutamate, which causes HT22 cell death by an oxytosis mechanism [24,25], a non-excitotoxic mechanism [24,26] where cell death is mediated by the depletion of intracellular glutathione (endogenous antioxidant) via the inhibition of the cystine/glutamate antiporter. In addition, J-147 has neurotrophic factor-like activity in a trophic factor withdrawal (TFW) cell assay; J-147 promotes the survival of freshly plated low-density cultured rat cortical neurons in F12/DMEM plus N2 medium, a serum-free culture medium frequently used for assaying neurotrophic factors [27]. In the preclinical in vitro development assays, the EC_50_ value for J-147-induced cell survival is 25 nM [16,17].

Because J-147 is a lead compound for further study to treat acute and chronic neurodegenerative diseases, it is important to determine if there are toxicities related to the compound. For this, we used the current industry standard, the CeeTox assay system which has recently been reviewed [23,28] and used extensively to assess the safety of drug candidates[23,29,30]. CeeTox analysis is an effective in vitro predictive toxicity screening assay to determine the toxicity profile of drugs being developed for human use [28,31] so that drug development can be derisked during early stages of development.

## Materials and Methods

### Neuroprotective compound

**Figure 1:**
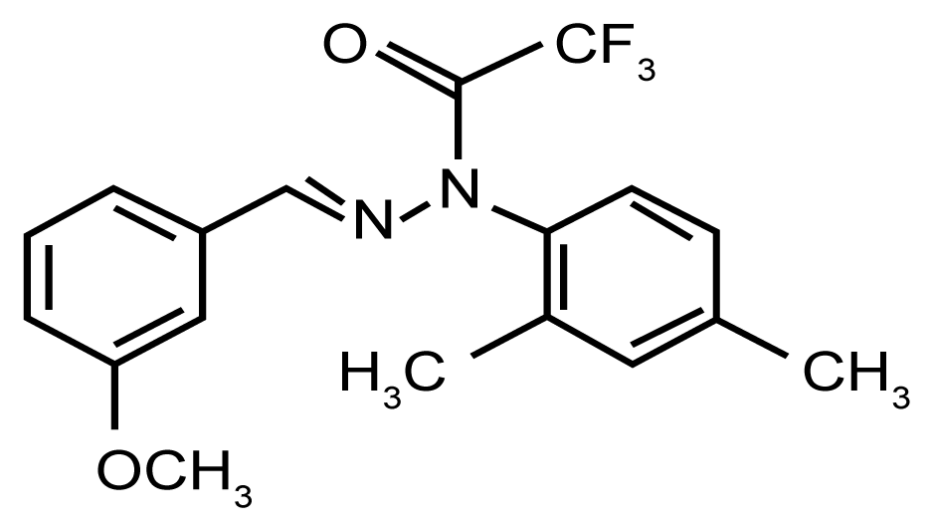
J-147 chemical structure.

Figure 1 presents the chemical structure of (E)-N-(2,4-Dimethylphenyl)-2,2,2-trifluoro-N′-(3-methoxybenzylidene) acetohydrazide (J-147), the lead compound being developed to treat neurodegenerative diseases.

### Experimental protocol

All Ceetox assays utilizing the rat hepatoma (H4IIE) cell line as the test system were conducted by CeeTox Inc Kalamazoo, MI. Ceetox assays are described in detail by McKim [28] and Lapchak [29, 30,32] and according to a Standard Operating Procedure (SOP). CeeTox Inc. did not have access to chemical structures or identities during assay.

#### Drug solubility

For solubility analysis, a stock solution in 100% DMSO was used to prepare dosing solutions of 1–300 μM in culture medium. The final concentration of DMSO in the 0–100 μM solutions was 0.5% and at the 300 μM solution DMSO was 1.5%. The final dosing solutions were prepared in Eagles Minimum Essential Medium. All experiments that used DMSO as the drug solvent also included a DMSO negative control. Negative controls of medium plus DMSO (0.5%) were included with and without cells. A positive control for complete cell death received 1 mM digitonin in medium on the day of dosing [28].

For solubility, the test compounds were prepared in DMSO and the appropriate amounts were then added to complete medium containing 10% bovine serum and 10% calf serum at 37°C. The samples were evaluated using light scattering with a Nepheloskan instrument. A reading that was greater than or equal to 3 times background was considered the limit of solubility [28].

#### H4IIE cell line

Rat hepatoma derived H4IIE cells were used as the test system because the cell have a rapid doubling time in culture (i.e. 22 hours) [28]. The culture medium used for these cells was Eagles Minimum Essential Medium (MEM) with 10% bovine serum and 10% calf serum (Invitrogen). H4IIE cells were seeded into 96-well plates and allowed to equilibrate for approximately 48 hr before drug assay to allow cells to move into a stable growth phase prior to treatment. Following the equilibration period the cells were exposed to J-147 at concentrations of 1–300 μM. Three to 7 replicates were done for each assay to construct concentration-response curves. Solubility was determined by Nephalometry techniques immediately after dosing and prior to harvesting the cells at 6 or 24 hr. Following the incubation period, cells or their supernatant (culture medium) were analyzed for changes in cell proliferation (cell mass), membrane leakage, mitochondrial function, oxidative stress, and apoptosis. The resultant exposure concentration response curves were graphed and analyzed for determining the concentration that produced a half maximal response or TC_50_ [28,30].

### General cellular measures of toxicity

#### Cell mass

Cell mass in each well was measured with a modified propidium iodide (PI) [33], a specific nucleic acid binding dye that fluoresces when intercalated within the nucleic acids. The 15nm shift enhances PI fluorescence approximately 20 times while the excitation maxima are shifted 30–40nm. Triton-X-100 was used to permeabilize the H4IIE cells thereby allowing the PI access to intracellular RNA and DNA. Fluorescence was measured using a Packard Fusion plate reader at 540 nm excitation and 610 nm emission [28]. Data is collected as relative fluorescent units (RFU) and expressed as percent change relative to control;

#### Membrane toxicity

The presence of α-Glutathione S-transferase (α-GST), an enzyme leakage marker, was measured in the culture medium using an ELISA assay purchased from Argutus Medical [28,34]. At the end of the exposure period, the medium covering the cells in each well was removed and stored at 80°C until assayed. Absorbance values were measured with a Packard SpectraCount^™^ reader at 450 nm and reference absorbance at 650 nm. Leakage of α-GST from the cell into the culture medium was determined by collecting the culture medium at the end of the exposure period. Thus, the values measured represent total enzyme leakage lost over the exposure period. *3-[4,5-dimethylthiazol-2-yl] 2,5-diphenyltetrazolium bromide (MTT)*: After the medium was removed from a plate for α-GST analysis, the cells remaining in each well were evaluated for their ability to reduce soluble-MTT (yellow) to formazan-MTT (purple) [28,35,36]. An MTT stock solution was prepared in complete medium just prior to use and warmed to 37°C. Once the medium was removed from all wells, MTT solution was added to each well and the plate was allowed to incubate at 37°C for 3–4 hr. Following incubation, all medium was removed and the purple formazan product was extracted using anhydrous isopropanol. Sample absorbance was read at 570 nm and reference absorbance at 650 nm with a Packard Fusion reader. The control for 100% dead or maximum enzyme release was based on cells treated with 1 mM digitonin at the time of dosing. Percent dead cells relative to digitonin treated cells was determined and then subtracted from 100% to yield the percent live cells.;

#### Cellular ATP content

Adenosine triphosphate (ATP) content was determined using a modification of a standard luciferin/luciferase luminescence assay [37] based on a reaction between ATP + D-luciferin + oxygen catalyzed by luciferase to yield Oxyluciferin + AMP + PPi + CO2 + light. The emitted light is proportional to the amount of ATP present [28]. At the end of the 24-hr exposure period the medium was removed from the cells and the ATP cell lysis buffer added to each well. Plates were analyzed immediately or stored at −20°C until needed. On the day of analysis, the plates were thawed and calibration curve prepared with ATP in the same liquid matrix as samples. ATP was quantified by adding ATP substrate solution and then reading luminescence on a Packard Fusion Luminescence reader. Normalized ATP content (pmoles ATP/million cells) in treated cells was extrapolated using the regression coefficients obtained from the linear regression analysis of the calibration curve. Background corrected luminescence was used to determine percent change relative to controls by dividing treated values by control values and multiplying by 100.

### Oxidative stress

#### Intracellular glutathione (GSH) levels

Intracellular glutathione levels were determined as described previously [29,32,38,30]. Briefly, the sulfhydryl group of GSH reacts with DTNB (5,5′-dithio-bis-2-nitrobenzoic acid, Ellman’s reagent) and produces a yellow colored 5-thio-2-nitrobenzoic acid (TNB). The mixed disulfide, GSTNB (between GSH and TNB) that is concomitantly produced, is reduced by glutathione reductase to recycle the GSH and produce more TNB. The rate of TNB production is directly proportional to the concentration of GSH in the sample. Measurement of the absorbance of TNB at 405 or 412 nm provides an accurate estimation of GSH in the sample. At the end of the exposure period, the medium was removed from the cells and metaphosphoric acid (MPA) was added to each well. Plates were then shaken for 5 min at room temperature and stored at −20°C until needed. The sample plates were thawed just prior to analysis and centrifuged at > 2000 × g for 2 min. Sample aliquots were removed and transferred to a clean 96-well plate along with appropriate standard curve controls. Sample pH was neutralized just prior to analysis and each well received an aliquot of PBS reaction buffer containing Ellman’s reagent, NADPH, and glutathione reductase. The plates were shaken for 15–30 min at room temperature and glutathione content was determined colorimetrically with a Packard Fusion reader at 415 nm. The assay is based on the concept that all GSH is oxidized to GSSG by DTNB reagent. Two molecules of GSH are required to make one molecule of GSSG (oxidized glutathione). Total GSH was determined by reducing GSSG to 2GSH with glutathione reductase. A standard curve was prepared with oxidized glutathione (GSSG) over a range of concentrations. These concentrations are then converted to glutathione equivalents (GSX) essentially by multiplying the GSSG standard concentrations by 2. The amount of GSX expressed as pmoles/well was determined using the standard curve and regression analysis and are expressed as percent of control;

#### Lipid peroxidation measured as 8-Isoprostane (8-ISO or 8-epi PGF2α)

8-ISO levels were determined using an ELISA (Cayman Chemical Inc). 8-ISO is a member of a family of eicosanoids produced nonenzymatically by random oxidation of tissue phospholipids by oxygen radicals. Therefore, an increase in 8-ISO is an indirect measure of increased lipid peroxidation [39]. At the end of the exposure period, plates were either analyzed immediately or stored at −80°C until needed for analysis. Color development, which is indirectly proportional to the amount of 8-ISO present in the sample, was read on a Packard Fusion or equivalent plate reader at 415 nm [28]. Background absorbance produced from Ellman’s reagent is subtracted from all wells. Non-specific binding is subtracted from the maximum binding wells to give a corrected maximum binding expressed as B_o_. The percent of bound (B) relative to maximum binding capacity (Bo) for all unknown samples and for standards was determined an expressed as (%B/Bo). The %B/Bo for standards was plotted against the log of 8-ISO added to yield the final standard curve. This curve was used to convert %B/Bo to pg 8-ISO/mL of sample.

### Apoptosis

Caspase 3 activity was determined using a caspase substrate (DEVD, Asp-Glu-Val-Asp) labeled with a fluorescent molecule, 7-Amino-4-methylcoumarin (AMC). Caspase 3 cleaves the tetrapeptide between D and AMC, thus releasing the fluorogenic green AMC [28]. Following the test article exposure to cells in 96-well plates, medium was aspirated from plates and PBS added to each well. Plates were stored at −80°C to lyse cells and store samples until further analysis. On the day of analysis, plates were removed from freezer and thawed. Caspase buffer with fluorescent substrate was added to each well and incubated at room temperature for 1hr. AMC release was measured in a spectrofluorometer at an excitation wavelength of 360 nm and an emission wavelength of 460 nm. Values are expressed as relative fluorescent units (RFU). After sample plates were completely thawed, the caspase substrate buffer mix was added to each plate. Plates were incubated at room temperature for 1hr, shielded from light. Plates were read using a in a spectrofluorometer at an excitation wavelength of 360 nm and an emission wavelength of 460 nm. Values were expressed as relative fluorescent units (RFU).

### P-glycoprotein (PgP) binding using the MTT assay

The H4IIE cells possess high levels of PgP protein in the outer membrane and be used effectively for evaluation of drug binding to PgP [28,40–42]. For this assay, cells are incubated with and without cyclosporin A (CSA) (a PgP inhibitor) at a single exposure concentration (50 μM) and the difference in toxicity determined with the MTT assay. Compounds with increased toxicity in the presence of CSA have a high probability of binding to PgP proteins. However, compounds of low toxicity will typically not show a difference relative to the addition of CSA, regardless of whether they bind to PgP. At the end of the 24-hr exposure period the culture medium was removed and the remaining attached cells were assayed for their ability to reduce MTT. Viable cells will have the greatest amount of MTT reduction and highest absorbance values. Percent control values were determined by dividing the mean absorbance/fluorescence of the treatment group by the mean absorbance of the control group and multiplying by 100.

### Metabolic stability

Metabolic stability was conducted using pooled microsomes from non-induced male Sprague-Dawley rats [28]. J-147 was incubated for 30 minutes at 37°C at concentrations of 1 μM. Subsequent HPLC analysis using a Waters Alliance 2795 in combination with a Waters Quattro Premier mass spectrometer measured disappearance of the parent molecule. The compounds were run on a Waters X-BridgeC18 (186003021) 50 × 2.1 mm column with 3.5 micron particle packing at a flow rate of 1 ml/min and with the temperature maintained at 50°C. Solvent A was water with 0.1% formic acid. Solvent B was acetonitrile with 0.07% formic acid. The data for 3 replicates are expressed as percent of parent remaining. Metabolism rankings are based on extent of metabolism with a percent remaining breakdown of: 100-65% = low; 65-45%=moderate; <45%=high.

### Genotoxicity studies

AMES test [43–45] studies were done using 2 strains of bacteria, specifically S. typhimurium TA989: hisD3052, rfa, uvrB/pKM101, which detects frameshift mutations and S. typhimurium TA100: hisG45, rfa, uvrB/pKM101, which detects base-pair substitutions either in the presence or absence of an S9 fraction from the livers of Aroclor 1254-treated [46, 47] rats. Under all conditions tested, there was no significant (≥2 fold) increase in the number of colonies formed in the presence of J-147 up to a concentration of 0.36 mM, clearly showing that J-147 was not mutagenic.

## Results

### CeeTox analysis

J-147 was soluble up to and including 300 μM in the culture medium system. Thus, the pharmacological effects of J-147 reported herein are not related to drug solubility.

#### Effects on cellular toxicity

**Figure 2:**
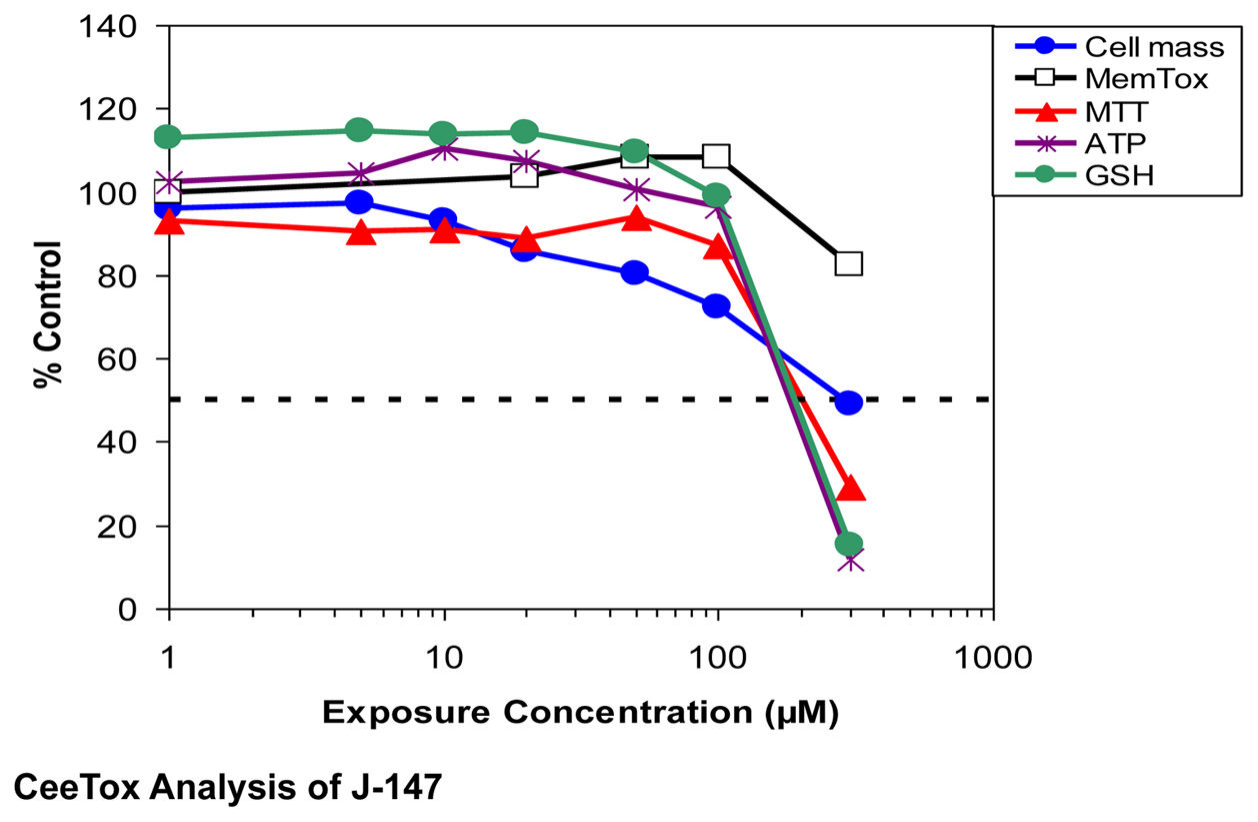
Effects of J-147 on cellular toxicity, oxidative stress and apoptosis following incubation of rat hepatoma derived H4IIE cells with the drug. Data is expressed as % control for cell mass (blue line), membrane toxicity (black line open square), MTT assay (red line), ATP (purple line), GSH content (green line) or pg/ml for 8-isoprostane. J-147 produced a significant decreases in cell proliferation between 20–100 μM, and both GSH and MTT were affected with TC_50_ values >200 μM.

Figure 2 shows dose-response profiles for the effects of J-147 on cellular toxicity using a 24hour analysis endpoint. For J-147 (Figure 2 and Table 1), the toxicity profile of J-147 was good without significant cell death (cell death TC_50_ = 293 μM) at any concentration tested in the assay. Most notable with J-147 was a decrease in cell proliferation between 20–100 μM that occurred prior to effects on the other markers and in the absence of cell death. The decrease in the mitochondrial markers (MTT and ATP) and total glutathione (GSH) at the highest exposure concentration (300 μM) was greater than the loss of cells, suggesting some effect of J-147 on mitochondria (Tables 1 and 2). As shown in Figure 2, other parameters had similar concentration-response curves in culture, with decreases in cellular ATP content (TC_50_ = 210 μM), and MTT reductase activity (TC_50_ = 228 μM).

#### Effects on oxidative stress markers

**Figure 3:**
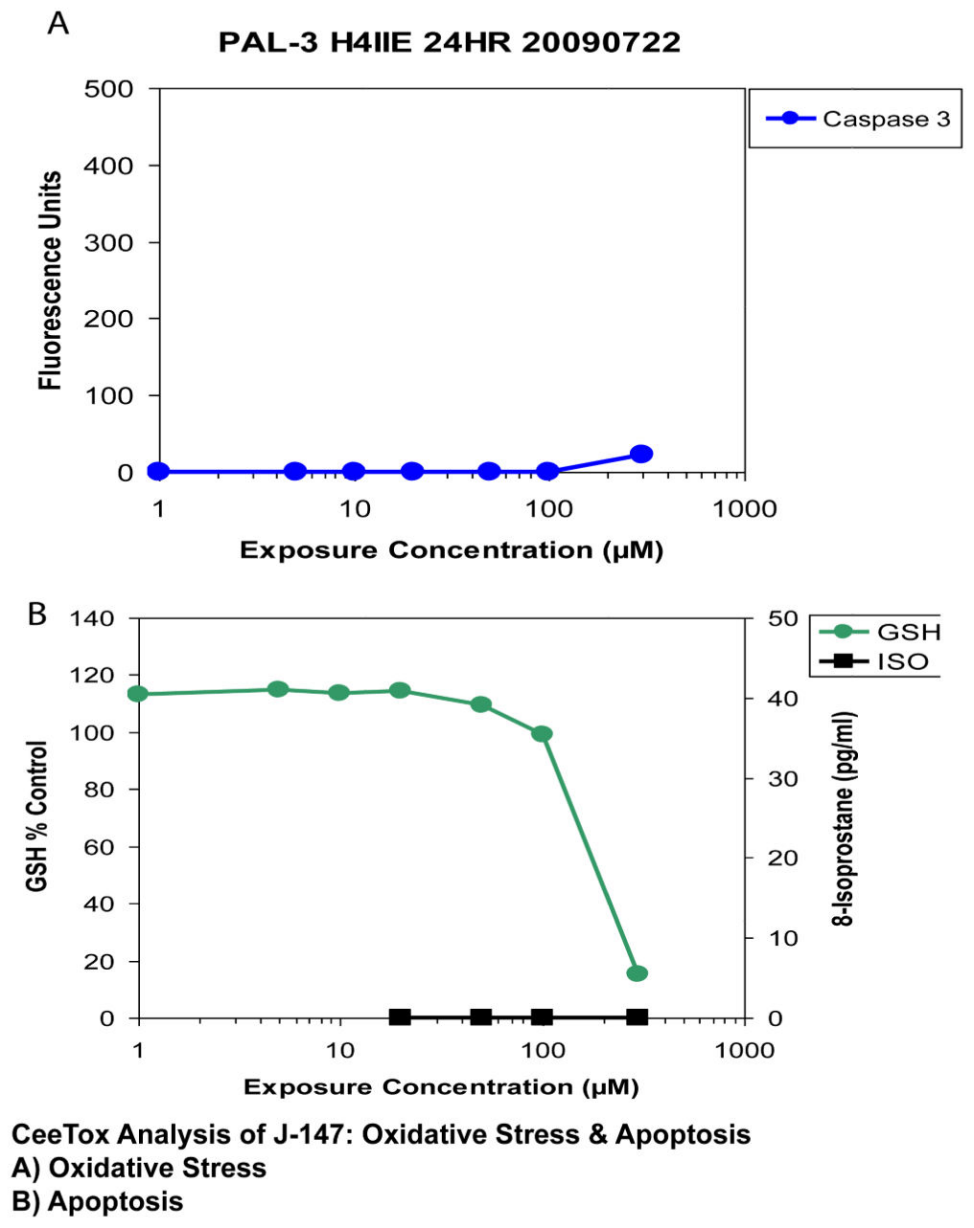
Effects of J-147 on (A) oxidative stress (8-isoprostane and GSH) and (B) apoptosis (caspase-3) following incubation of rat hepatoma derived H4IIE cells with the drug. Data is expressed as % control for isoprostane (black line), GSH (green line), and caspase 3 (blue line). J-147 produced a significant decrease in GSH with a TC_50_ value of 217 μM.

Figures 3A show a comparison of 2 markers used to determine the effects of J-147 on markers of oxidative stress. For this measure, we used intracellular GSH content and lipid peroxidation measured as 8-isoprostane [28]. J-147 reduced intracellular GSH content with a TC_50_ = 217 μM, but did not have any effect on 8-isoprostane.

#### Effect on apoptosis or caspase-3 activity

As a measure of the effects of the study drugs on apoptotic mechanisms, we used caspase-3 activity [28], which is a key mediator of apoptosis in neuronal cells, but also has non-apoptotic functions [48]. J-147 did not affect caspase-3 activity (Figure 3B).

#### Drug P-glycoprotein (PgP) binding

Table 3 shows that J-147 had a low interaction with the Permeability-glycoprotein when cells are incubated with and without cyclosporin A (CSA), a PgP inhibitor at a single exposure concentration (50 μM). Compounds with increased toxicity in the presence of CSA have a high probability of binding to PgP proteins. However, compounds of low toxicity will typically not show a difference relative to the addition of CSA, regardless of whether they bind to PgP.

#### Metabolic stability of J-147

J-147 was metabolically unstable, that is it was highly metabolized with only 12% of the parent compound remaining after incubation with rat microsomes (phase 1 metabolism) for 30 minutes at 37°C measured using HPLC/MS. It is important to note that H4IIE cells have low constitutive metabolic activity, but is sensitive to CYP1A1/2 induction. Compounds that induce CYP1A enzymes may also be substrates and undergo metabolism in the H4IIE system.

#### C_Tox_ ranking

**Figure 4:**
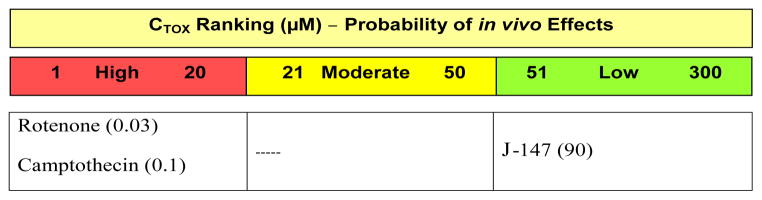
C_Tox_ Ranking Profile CTox ranking values for toxic control compounds and J-147 are provided in parentheses. Metabolic Stability

For each compound studied, a C_Tox_ value was generated by CeeTox Inc., using a patented proprietary algorithm [49]. C_Tox_ values were generated from the TC_50_ values in this report. The C_Tox_ ranking for J-147, which is an estimate of a sustained concentration expected or necessary to produce toxicity in a rat 14 day repeat dose study was 90 μM. To validate the CeeTox assay, rotenone [50,51], a mitochondrial inhibitor that directly interferes with electron transport chain to inhibit ATP synthesis was used as well as camptothecin [52–54], a cytotoxic quinoline alkaloid which inhibits the DNA enzyme topoisomerase I were used. The C_Tox_ ranking for rotenone and camptothecin were 0.03 and 0.1 μM, respectively (Figure 4).

### Genotoxicity studies

AMES test [43–45] studies were done using 2 strains of bacteria, specifically *S. typhimurium* TA989: hisD3052, rfa, uvrB/pKM101, which detects frameshift mutations and *S. typhimurium* TA100: hisG45, rfa, uvrB/pKM101, which detects base-pair substitutions either in the presence or absence of an S9 fraction from the livers of Aroclor 1254-treated [46,47] rats clearly showed that J-147 was mutagenic. Under all conditions tested, there was no significant (≥2 fold) increase in the number of colonies formed in the presence of J-147 up to a concentration of 0.36 mM.

## Discussion

The present study determined the pharmacological profile of J-147, a lead neurotrophic/neuroprotective compound currently being developed for the treatment of a variety of neurodegenerative diseases. The neuroprotective treatment of acute and chronic neurodegenerative diseases has been formidable challenge for over 50 years; a time period when many novel strategies have failed in clinical trials. There are many reasons for the failure of neuroprotectives that have been tested in clinical trials, primarily lack of effect, lack of long-term or reproducible efficacy, or dose-limiting toxicity that has prevented further development of promising candidates

In this study, we used the CeeTox screening panel to de-risk the further development of the drug to ensure lack of severe adverse events or toxicities [23,28–30]. The C_Tox_ value or sustained concentration expected to produce toxicity in a rat 14 day repeat dose study was estimated to be 90 μM. The C_Tox_ value suggests that there is a low probability of in vivo toxicity effects related to chronic long-term administration of J-147, but there are numerous caveats associated with the interpretation of the CeeTox panel assay, especially the fact that the C_Tox_ value is for 14 day repeat dosing. The general CeeTox cellular health panel suggests that J-147 produces some adverse effects, but only with very high concentrations of the drug, where TC_50_ values was >275 μM. However, one must note that with drug exposures in the range of 40–50 μM, none of the measures showed a significant change compared to control. From a drug development perspective, it is interesting to note that J-147 is efficacious in vitro using HT22 cells and primary cortical cells with an EC_50_ values in the range of 0.06–0.115 μM [17] suggesting that there is a significant therapeutic safety window of 782.6–1500 fold based upon efficacy/toxicity ratios.

In H4IIE cells, there was a notable decrease in cell proliferation between 20–100 μM that occurred prior to effects on the other markers that were measured and in the absence of cell death. The decrease in the mitochondrial markers (MTT and ATP) and total glutathione (GSH) at the highest exposure concentration (300 μM) was greater than the loss of cells, suggesting an effect of J-147 on mitochondria. Taken together, it appears that there are dose-related effects of J-147 on H4IIE cells, in particular on mitochondrial function. Nevertheless, a recent mouse AD study suggested that chronic J-147 administration was safe and beneficial to reduce memory impairment associated with the particular AD mouse that was studied [17].

The metabolic stability of 1 μM J-147 was measured using a male Sprague-Dawley rat microsomal preparation. The assay measured disappearance of the parent molecule, but did not measure any of the known metabolites of J-147 [55]. J-147 was metabolically unstable (highly metabolized) with only 12% of parent remaining after incubation with rat microsomes (phase 1 metabolism). The H4IIE cell model has low constitutive metabolic activity and, with some exceptions, the calculated C_Tox_ value reflects toxicity associated with the non-metabolized form of the test article. It is important to note that rat H4IIE constitutively express several key cytochrome P(CYP) 450 enzymes including CYP1A, CYP2B, CYP2C, and CYP3A activity in the H4IIE cells. In addition, there is both glucuronide and glutathione conjugation capability and the H4IIE cell line used in this study is sensitive to inducers of CYP1A [28]. In most instances, the results obtained with H4IIE cells reflect “toxicity” due to the non-metabolized forms of the test compounds. However, it cannot be excluded that situations may occur where the test compound both induces CYP and is also metabolized by one of the constitutively expressed CYP isoforms, thus, toxicity may be the result of metabolism and/or conjugation.

Recent studies have determined that there at least 5 pharmacologically active metabolites of J-147, designated M1-M5 [55]. Of the 5 metabolites, M1-M3 have been shown to be neuroprotective in an oxidative stress assay and neurotrophic in a trophic factor withdrawal assay [55]. Because they retain some level of neuroprotective or neurotrophic activity, complete CeeTox analysis should be conducted on M1-M5 to ensure a reasonable safety profile. Moreover, as recently reported by Prior et al. [17], although none of the compounds are chemically similar to monoamine oxidase B (MAOB) inhibitors or dopamine reuptake inhibitors [17], the finding that J-147 can inhibit MAOB and the dopamine transporter with EC_50_ values of 1.88 μM and 0.65 μM, respectively, suggests that additional preclinical safety studies are warranted.

In conclusion, CeeTox analysis proved useful as a de-risking tool and provided useful information concerning the in vitro concentration where cellular toxicity may occur, compared to the neuroprotection profile of the drug. The observation that the C_Tox_ value for J-147 is 90 μM indicates that J-147 should continue to be developed as a lead compound, but additional toxicity and pharmacology studies are recommended.

## Figures and Tables

**Table 1:** Comparison of J-147 with Control compounds on Cell Toxicity.

Compound	Cell Number TC_50_ (μM)	Mem Tox TC_50_ (μM)	MTT TC_50_ (μM)	ATP TC_50_ (μM)	Predicted C_tox_ (μM)
J-147	293	>300	228	210	90
Rotenone	0.04	0.93	0.07	0.05	Control (~ 0.03)
Camptothecin	0.6	>300	0.5	0.3	Control (~ 0.1)

TC_50_=concentration that produced a half-maximal response.

Cell Number (or mass). (n=7 replicates).

MemTox=Membrane toxicity measured using GST leakage. (n=3 replicates).

MTT=3-[4,5-dimethylthiazol-2-yl]-2,5-diphenyltetrazolium bromide. (n=7 replicates).

ATP=adenosine triphosphate content. (n=7 replicates).

**Table 2:** Comparison of J-147 with Control compounds on Oxidative Stress and Apoptotic Cell death.

Compound	Total GSH TC_50_ (μM)	Percent Change in Total GSH	Membrane Lipid Peroxidation	Caspase 3 Activity (Index/Dose)
J-147	217	−84.9	0	NC
Rotenone	0.3	−100.0	0	NC
Camptothecin	3.9	−64.5	1	3/1

Glutathione (GSH) Data:

Decrease in Total GSH indicated by (−).

Increase in Total GSH indicated by (+).

Membrane Lipid Peroxidation Data:

0=No Change,

1=Modest increase with maximum values<15 pg/mL,

2=Concentration related increase with maximum values>15 pg/mL,

3=Concentration related increase with a maximum value>30 pg/mL.

Caspase 3 Data:

0–200=1, 200–400=2, 400–600=3, 600–800=4, 800–1000=5, 1000–1200=6, 1200–1400=7, 1400–1600=8, 1600–1800=9, 1800–2000=10.

**Table 3:** Drug P-glycoprotein (PgP) Binding.

Compound	% Control (Compound)	% Control (Compound + CSA)	% Difference
J-147	93.7	92.1	NC
Rotenone	80.5	86.1	NC
Camptothecin	76.6	85.3	NC

PgP Interaction Ranking (based on % Difference in the absence and presence of CSA, n=7 replicates). NC=No change.

**Table 4:** Microsomal Metabolic Stability.

Metabolic Stability		
Compound (1μM)	% Remaining	Metabolism Ranking
J-147	12	High
Reference Compounds		
Midazolam	0–3	High
Terfenadine	6–23	High

Metabolic stability was conducted using pooled microsomes from non-induced male rat (Sprague-Dawley) animals. The test compounds were incubated for 30 minutes at 37°C at concentrations of 1 μM. Subsequent LC/MS analysis measured disappearance of the parent molecule. The data are expressed as percent of parent remaining. Reference compounds were included for highly metabolized compounds for comparison. Metabolism rankings are based on extent of metabolism with a percent remaining breakdown as follows: 100-65= low; 65-45=moderate; <45=high.
